# Synthetic Strategies for Bioactive Oligosaccharides

**DOI:** 10.3390/biom15121691

**Published:** 2025-12-04

**Authors:** Jing Liu, Wenyuan Fang

**Affiliations:** College of Pharmacy, Jining Medical University, Rizhao 276826, China; liujingll@mail.jnmc.edu.cn

**Keywords:** oligosaccharides, chemical synthesis, one-pot synthesis, preactivation strategy

## Abstract

Carbohydrates are essential constituents of numerous biological systems, playing key roles in fundamental processes such as cellular recognition and immunological responses, while also offering significant potential in medical diagnostics and pharmaceutical development. However, the structural complexity of naturally occurring carbohydrates-characterized by heterogeneous glycosylation patterns and diverse branching architectures-poses considerable challenges in the isolation and preparation of homogeneous oligosaccharide samples. Recent advances in synthetic chemistry have led to substantial progress in carbohydrate synthesis, with modern glycosylation methodologies achieving improved stereochemical control and enhanced reaction efficiency, thereby enabling the precise and programmable construction of biologically relevant oligosaccharides. This review provides a systematic evaluation of six major strategies for oligosaccharide assembly: One-pot synthesis strategy, orthoganal protection strategy, preactivation strategy, linear and convergent block strategy, programmable one-pot synthesis strategy, and solid-phase synthesis strategy. For each strategy, we examine the key technological innovations, representative applications, and current limitations. Furthermore, the review discusses emerging trends in the field, emphasizing the transformative role of intelligent automation and machine learning in accelerating the discovery and synthesis of complex carbohydrates.

## 1. Introduction

**Figure 1:**
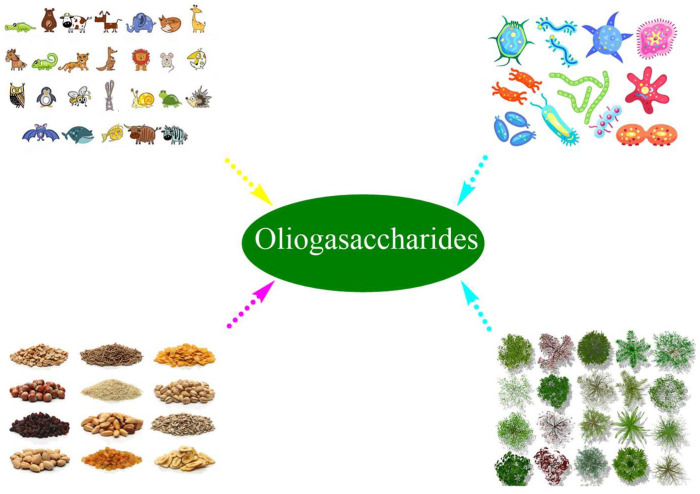
Origin of oligosaccharides. These carbohydrate chains are widely distributed across botanical, zoological, and microbial sources [1,2].

Oligosaccharides are carbohydrate polymers composed of 3 to 10 monosaccharide units linked by α- or β-glycosidic bonds, and they may adopt either linear or branched structures. These biomolecules are widely distributed across biological systems, including plant sources (e.g., cellulose-derived oligosaccharides), animal tissues (e.g., membrane-bound glycosphingolipids), and microorganisms (e.g., bacterial lipopolysaccharides), as depicted in Figure 1 [1,2]. As complex biological macromolecules, oligosaccharides play essential roles in regulating physiological processes [3,4,5,6]. For instance, sulfated glycosaminoglycans such as chondroitin sulfate proteoglycans are involved in neural tissue development and repair [1], while linear polymers like hyaluronan contribute to cellular hydration and epidermal regeneration [2]. Moreover, the structural stability and functional activity of glycoproteins often depend on *O*-linked and *N*-linked oligosaccharide chains, exemplified by human follicle-stimulating hormone [3]. Oligosaccharides also exhibit diverse therapeutic potentials in multiple disease contexts, including cancer targeting (e.g., Globo-H hexasaccharide as a tumor-associated antigen), microbial inhibition (e.g., blockade of bacterial adhesion via modified lipopolysaccharides), inflammation modulation (e.g., heparan sulfate derivatives), and antiviral defense (e.g., sialylated glycans that interfere with pathogen binding). These properties underscore their significance as promising candidates for pharmaceutical development [7,8].

The human body harbors over 300 genes involved in glycan biosynthesis, the expression and activity of which are tightly regulated through a variety of molecular mechanisms. This intricate regulatory system leads to the formation of a wide variety of structurally distinct glycoconjugates, including glycoproteins and glycolipids [4]. Although these biomolecules perform essential biological functions, the isolation of homogeneous oligosaccharides from natural sources remains technically challenging and economically impractical due to inherent structural heterogeneity. As a result, synthetic chemistry has emerged as a critical approach for generating well-defined carbohydrate structures, allowing for comprehensive investigations into their biological functions and supporting the advancement of therapies based on glycans [5]. However, the construction of glycosidic linkages continues to pose significant challenges, particularly in achieving high regio- and stereoselectivity-especially in the formation of α-sialosides and β-mannosides-while minimizing side reactions. Recent advances in synthetic methodologies have substantially addressed these limitations, leading to significant advancements in carbohydrate synthesis research. This section provides an overview of these key strategies, focusing on their underlying principles, practical applications, and recent innovations.

## 2. Chemical Synthesis Strategy of Oligosaccharides

The primary objective of oligosaccharide synthesis is to construct glycosidic bonds with precise regio- and stereochemical control, while simultaneously enhancing synthetic efficiency and streamlining purification procedures. The following sections examine six major synthetic strategies, highlighting their methodological developments and representative applications.

### 2.1. One-Pot Synthesis Strategy

One-pot synthesis represents a transformative advancement in oligosaccharide chemistry, enabling the sequential assembly of glycosyl donors and acceptors within one pot without the need for intermediate isolation [9]. This approach significantly improves synthetic efficiency by minimizing the need for protective group adjustments and purification steps [10,11], while ensuring consistent product uniformity [9,12,13,14,15,16]. The progress in this field has been driven by two key innovations: the development of high-performance glycosyl building blocks and the successful resolution of stereochemical challenges in complex glycosylation reactions, including α-sialylation and β-mannosylation.

Key challenges and technical breakthroughs. The implementation of one-pot synthesis was initially hindered by significant technical obstacles, primarily reflected in two critical limitations: (1) the absence of dependable methods for stereoselective α-sialylation, which is essential for the construction of sialylated oligosaccharides involved in viral recognition, and (2) the difficulty in establishing efficient β-mannosylation pathways, due to the thermodynamic preference for α-configuration formation resulting from anomeric stabilization effects [17,18].

To address the challenges associated with α-sialylation, Takahashi’s research group introduced the 4-*O*, 5-*N*-oxazolidinone protecting group, which stabilizes the transition state via intramolecular hydrogen bonding, thereby promoting selective α-anomer formation during glycosylation reactions [17]. Subsequent work by Crich’s team further advanced this strategy through structural optimization, developing an *N*-acetylated oxazolidinone derivative that exhibits enhanced stereocontrol and compatibility with tandem synthetic protocols [19]. Nevertheless, the inherently low reactivity of sialyl donors remains a limiting factor in their application to multistep, one-pot synthetic sequences. Recent progress has been achieved through the design of adamantane-functionalized donor systems in conjunction with *N*-acyloxazolidinone protecting groups synergistic approach that significantly enhances donor reactivity while preserving high stereoselectivity [20].

In β-mannosylation strategies, Crich’s research group developed a methodology utilizing 4,6-benzylidene protecting groups; this structural feature imposes conformational constraints on the mannosyl donor, thereby favoring an S_N_2-type displacement mechanism and enhancing β-anomeric selectivity [18]. More recently, Sasaki’s team has introduced an innovative approach employing 2,6-lactone-based glycosyl donors, in which the cyclic ester moiety acts as an intrinsic stereodirecting element to facilitate the formation of β-mannosidic linkages [21]. These methodological advances have enabled the integration of α-sialylation and β-mannosylation reactions within one pot synthesis, thus expanding the scope of accessible complex oligosaccharide architectures.

Implementation approaches. Contemporary one-pot glycosylation protocols are typically carried out using three distinct methodologies, each based on unique operational principles to control reaction dynamics and stereoselectivity.

Anomeric reactivity modulation approach: This methodology entails modulating the chemical reactivity at the anomeric center of carbohydrate donors through strategic manipulation of protective groups [22,23]. Building upon Fraser-Reid’s foundational “armed/disarmed” concept [22], it has been established that ether-type protecting groups (e.g., benzyl) generate “armed” donors with heightened reactivity, whereas ester-type protections (e.g., acetyl) result in “disarmed” donors exhibiting reduced reactivity [22]. In the context of bifunctional donor-acceptor systems, more reactive glycosyl donors selectively engage with acceptor hydroxyl groups, thereby enabling the stepwise and controlled assembly of oligosaccharides via reactivity-gradient-driven strategies [23]. This principle was effectively demonstrated by multiple research teams, which achieved the systematic construction of structurally diverse carbohydrate architectures through the sequential employment of sugar building blocks with progressively decreasing reactivity [23,24,25,26].

Orthogonal protective methodology: This approach utilizes glycosyl donors equipped with distinct leaving groups that require specific catalytic conditions, enabling selective activation of individual components in multicomponent donor systems [27]. Experimental findings from Ogawa’s group demonstrate the feasibility of this strategy: Thiophenyl glycosyl donors are efficiently activated under NIS-TfOH/AgOTf conditions, whereas fluoroglycosyl donors remain inert under the same conditions. In contrast, fluoroglycosyl donors exhibit reactivity when exposed to Cp_2_HfCl_2_-AgClO_4_, without triggering undesired reactions in thiophenyl analogs [27]. These mutually orthogonal activation profiles allow for programmable and sequential glycosylation, as demonstrated by the successful synthesis of complex, branched carbohydrate structures through controlled chain extension [7,28].

Preactivation methodology: This approach involves the initial activation of the glycosyl donor through a promoter before the addition of the glycosyl acceptor, leading to the creation of a stabilized reactive intermediate. The efficient formation of glycosidic bonds is achieved through the subsequent addition of the acceptor [29]. By decoupling the activation and coupling steps, this method minimizes potential interference among reaction components and allows for one-pot synthetic strategies regardless of anomeric reactivity differences [29]. The research group led by Van der Marel successfully applied this methodology to reducing sugars and thioglycoside derivatives, achieving efficient synthesis of linear oligosaccharide architectures [30].

**Figure 2:**
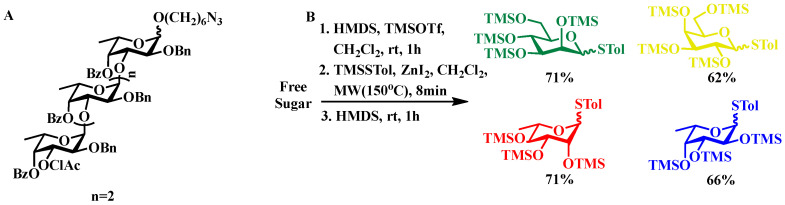
(**A**) Molecular structure of the characteristic tetrasaccharide repeating unit in type I fucoidan. (**B**) A one pot microwave-assisted synthetic protocol for fully silylated thioglycoside derivatives, in which microwave irradiation facilitates the rapid formation of silylated intermediates, thereby substantially reducing reaction time and improving overall synthetic efficiency.

Notable Implementations. Fucoidan segment construction: Liu’s research group achieved the construction of trisaccharides under mild reaction conditions using NIS/TMSOTf as activating agents and n-hexynyl benzoate glycosyl donors. This methodology was further expanded to the synthesis of tetrafucoside units—key structural motifs in type I fucoidan—through the use of phthaloyl-protected glycosyl donors (Figure 2A) [31].

Zhang et al. developed the OptiMer platform, which utilizes a curated repository of thioglycoside donors with predefined relative reactivity values. This platform establishes a correlation between donor activation potential and the anomeric proton NMR chemical shifts in acceptors, enabling automated design of synthetic pathways. Subsequently, Wong’s research group further advanced this approach by developing Auto-CHO software (FileMaker Pro 4.0), which facilitates complex, multistage syntheses, including the efficient synthesis of Globo-H hexasaccharide-a promising candidate for prostate cancer immunotherapy [32].

Microwave-enhanced synthetic strategies: Ko’s research team employed microwave-assisted methodology to efficiently generate transient per-*O*-trimethylsilylated sugar intermediates activated by TMSOTf. The subsequent reaction using methyl(4-methylphenylthio)silane under zinc iodide catalysis facilitated the synthesis of diverse 1,6-anhydrosugars and thioglycoside derivatives. These intermediates were further subjected to site-selective protection modifications, yielding precisely tailored molecular building blocks (Figure 2B).

**Figure 3:**
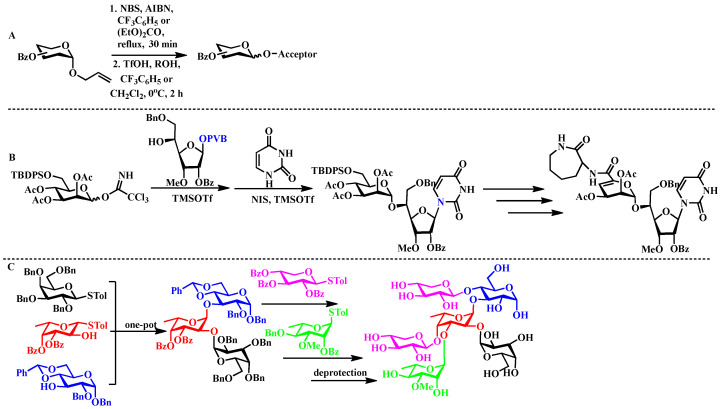
(**A**) TfOH-mediated one-step glycosylation of allyl glycosyl derivatives; (**B**) Synthesis of capuramycin via orthogonal sequential glycosylation: PVB-protected glycosyl donors undergo selective activation for nucleoside–glycan conjugation; (**C**) Regioselective assembly of the ATCV-1-associated hexasaccharide enabled by the Ye research group’s optimized stepwise addition protocol.

Das and colleagues developed an innovative method for allyl glycoside glycosylation that eliminates the requirement for carbon tetrachloride used as a solvent. The methodology utilizes silver trifluoromethanesulfonate as an activator in a mixed solvent system composed of diethyl carbonate and benzotrifluoride. This strategy integrates allylic halogenation with subsequent glycosylation into a single reaction vessel, thereby providing a highly effective pathway for the preparation of xylopyranoside di- and trisaccharide frameworks (Figure 3A) [33]. In the context of anti-tuberculosis drug development, the research group led by Carthy introduced a streamlined one-pot glycosylation protocol employing PVB-protected glycosyl donors. Their approach enables the efficient assembly of Capuramycin through sequential coupling of carbohydrate and nucleoside moieties within a unified reaction system (Figure 3B) [34]. In the field of virology-related carbohydrate synthesis, Ye and coworkers systematically optimized the stepwise addition of saccharide building blocks. Through rigorous parameter refinement, they achieved precise control over both regioselectivity and stereoselectivity, successfully accomplishing the one-pot synthesis of the structurally complex hexasaccharide segment associated with ATCV-1 viral capsid proteins (Figure 3C) [35].

### 2.2. Orthogonal Protection Strategy

This methodology involves the selective activation of glycosyl donors containing chemically orthogonal leaving groups or protecting groups under distinct reaction conditions, enabling the sequential formation of glycosidic bonds with high stereochemical precision [27,36]. It is particularly effective for the assembly of complex branched oligosaccharide structures, allowing for the stepwise coupling of multiple donors to a common acceptor while preserving reaction specificity. This method proves particularly beneficial when synthesizing molecular structures with multiple branching points, where conventional strategies may lead to undesired cross-activation side reactions.

The concept of orthogonal protection relies on the deliberate choice of both leaving groups and protecting groups that display distinct reactivities toward specific activation conditions. A representative example is glycosyl trichloroacetimidates, which can be activated under mild Lewis acid catalysis (e.g., TMSOTf), whereas thioglycosides remain stable under these conditions and require more potent electrophilic activators, such as NIS/TfOH or DMTST [27,36]. Glycosyl bromides and glycosyl fluorides exhibit different activation profiles-glycosyl bromides respond to AgOTf, while glycosyl fluorides necessitate more complex systems such as HfCp_2_Cl_2_/AgOTf [36]. Furthermore, fine-tuning of reactivity can be achieved through structural modifications of the leaving groups, for instance by varying sulfur-containing substituents (e.g., SEt vs. SPh), thereby enabling precise control within a hierarchical activation framework [36].

This approach enables flexible, sequential donor activation independent of inherent chemical reactivity, thereby offering exceptional versatility in the assembly of intricate molecular [7,28].

In the domain of specialized carbohydrate synthesis and polysaccharide assembly, orthogonally protected building blocks function as key intermediates in the preparation of structurally diverse monosaccharides, including deoxyamino sugars and microbial glycoconjugates [37,38,39,40,41,42]. These complex biomolecules are critically involved in elucidating microbial pathogenicity mechanisms and facilitating the development of immunotherapeutic and prophylactic agents. Their application is particularly significant in studies of host–pathogen interactions and vaccine design. The use of multi-level protection strategies allows for precise, stepwise manipulation of reactive sites, which is essential for the controlled construction of biologically relevant carbohydrate structures.

**Figure 4:**
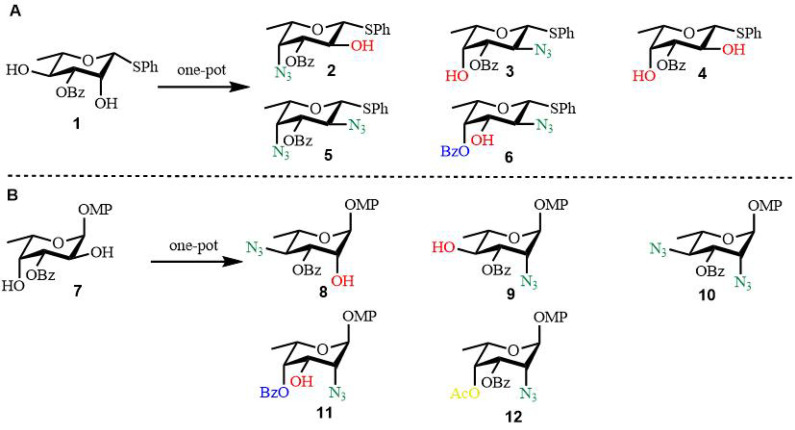
One-pot conversion of (**A**) L-rhamnose and (**B**) L-fucose into rare L-amino sugar. Reagents and conditions: Compounds **2**–**6** (2) Tf_2_O, Py, CH_2_Cl_2_, 0 °C, 20 min; TBANO_2_ (3 equiv), CH_3_CN, 0 °C, 2 h; TBAN_3_, rt, 2 h; 52% over 3 steps; (3) Tf_2_O, Py, CH_2_Cl_2_; TBAN_3_ (1 equiv), CH_3_CN, 0 °C, 1 h; TBANO_2_, rt, 2 h; 57% over 3 steps; (4) Tf_2_O, Py, CH_2_Cl_2_; TBANO_2_, CH_3_CN, rt, 8 h; 50% over 2 steps; (5) Tf_2_O, Py, CH_2_Cl_2_; NaN_3_, DMF; 74% over 2 steps; (6) Tf_2_O, Py, CH_2_Cl_2_; TBAN_3_ (1 equiv), CH_3_CN, 0 °C, 1 h; CH_2_Cl_2_:H_2_O(9:1), 80 °C, 10 h; 59% over 3 steps. Compounds **8**–**12** (8) Tf_2_O, Py, CH_2_Cl_2_; TBAN_3_ (1 equiv), CH_3_CN, 0 °C, 2 h; TBANO_2_, 60 °C, 5 h; 52% over 3 steps; (9) Tf_2_O, Py, CH_2_Cl_2_; TBANO_2_ (3 equiv), CH_3_CN, 0 °C, 1 h; NaN_3_, HMPA, 110 °C,10 h; 58% over 3 steps; (10) Tf_2_O, Py, CH_2_Cl_2_; NaN_3_, HMPA, 110 °C,10 h; 69% over 2 steps; (11) Tf_2_O (3 equiv), Me_2_SnCl_2_, 2,6-Lutidine, CH_2_Cl_2_, 0 °C; NaN_3_, HMPA, 110 °C,10 h; 49% over 2 steps; (12) Tf_2_O (3 equiv), Me_2_SnCl_2_, 2,6-Lutidine, CH_2_Cl_2_, 0 °C, 0.5 h; Ac_2_O, 2h; NaN_3_, HMPA, 110 °C,10 h; 52% over 3 steps.

Synthesis of bacterial oligosaccharides: Kulkarni and colleagues developed robust methodologies for the conversion of L-rhamnose and L-fucose into rare L-deoxyamino sugars through selective protection strategies [43]. In a key transformation, the β-L-rhamnothioglycoside 2,4-diol (compound **1**) underwent bistriflation, followed by regioselective S_N_2 substitution at both the axial C2 and equatorial C4 triflate positions using a range of nucleophilic reagents [44]. This efficient, single-reaction protocol enabled the synthesis of multiple L-fuco-configured sugar derivatives (compounds **2**–**6**), as depicted in Figure 4A [43]. Analogous transformations were carried out starting from the L-fucosyl 2,4-diol precursor (compound **7**), wherein sequential triflate displacement yielded L-rhamno-configured analogs (compounds **8**–**12**), illustrated in Figure 4B [43]. The resulting synthetic intermediates facilitated the preparation of biologically significant carbohydrate structures, including the repeating tetrasaccharide unit of the *O*-polysaccharide derived from Yersinia enterocolitica O:50 strain 3229, along with the trisaccharide component of the *O*-polysaccharide from Pseudomonas chlororaphis subsp. aureofaciens M7143 [43].

**Figure 5:**
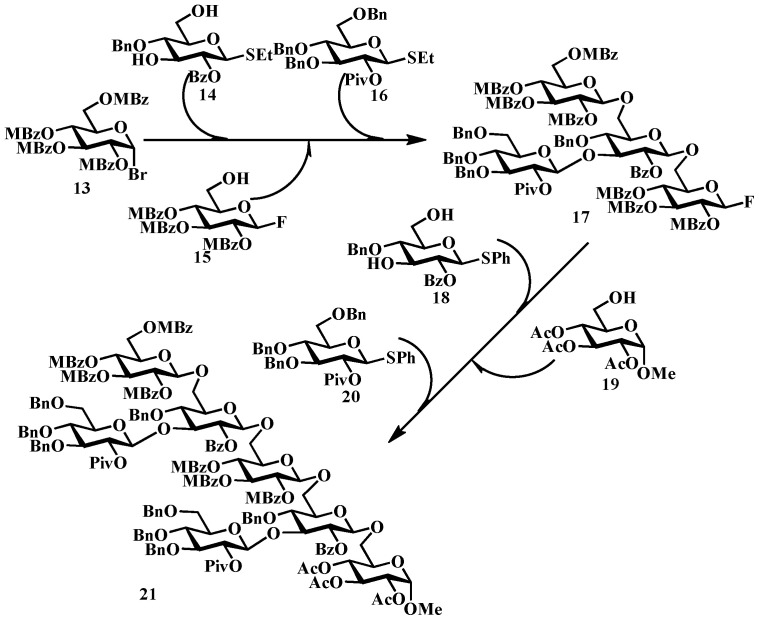
Takahashi’s one-pot synthesis of heptasaccharide. Reagents and conditions: Compound **17**, **13**, **14**, AgOTf, CH_2_Cl_2_, −20 °C; **15**, MeOTf, CH_2_Cl_2_, rt; **16** (1.80 equiv), CH_2_Cl_2_, rt. Compound **21**, **18**, HfCp_2_Cl_2_/AgOTf, CH_2_Cl_2_, 0 °C; **19** (1.25 equiv), DMTST (12.0 equiv), CH_2_Cl_2_, 0 °C; **20**, CH_2_Cl_2_, 0 °C.

Takahashi’s group demonstrated the effectiveness of orthogonal protection strategies in synthesizing a bioactive heptasaccharide that triggers phytoalexin-elicitor activity, accomplished through a one-pot sequence comprising six consecutive glycosylation steps [45]. The methodology utilized a sequential activation strategy for structurally diverse glycosyl donors: bromide **13** (activated by AgOTf), ethylthio derivatives **14**, **16**, and **18** (activated by MeOTf), fluoride **15** (activated by HfCp_2_Cl_2_/AgOTf), and phenylthio compound **20** (activated by DMTST), as depicted in Figure 5 [45]. The optimized synthetic protocol enabled the construction of the target molecule in a cumulative yield of 24%, representing one of the most complex one-pot glycosylation sequences reported to date [45].

### 2.3. Preactivation Strategy

The preactivation methodology provides an alternative strategy for one-pot synthetic approaches. In this approach, the glycosyl donor is activated through a stoichiometric promoter-mediated reaction before the glycosyl acceptor is introduced, generating a stabilized reactive intermediate, such as a glycosyl oxocarbenium ion [29,46]. The subsequent addition of the acceptor enables efficient glycosidic bond formation [29], thereby temporally separating the activation and coupling steps. This separation effectively circumvents potential cross-reactivity between donor and acceptor, allowing for high-efficiency one-pot synthesis regardless of differences in anomeric reactivity and enabling the use of building blocks with identical aglycone moieties [29,30]. The distinct temporal control over activation and coupling enhances overall reaction precision, thereby broadening the range of compatible substrates in oligosaccharide assembly.

The preactivation strategy offers distinct advantages over alternative methodologies. In comparison with reactivity-dependent one-pot synthesis and orthogonal protection approaches, it presents two primary benefits. First, it eliminates the need for reactivity tuning: unlike methods that rely on precise modulation of protecting groups to control donor reactivity, preactivation employs glycosyl donors with identical aglycone structures, thereby simplifying the synthesis of building blocks [29,46,47]. Second, it enables a more streamlined molecular design: in contrast to orthogonal protection strategies that require diverse leaving groups, preactivation uses uniform activating groups, reducing synthetic complexity. However, this approach requires stoichiometric amounts of activator to ensure complete donor activation and to suppress potential side reactions, such as spontaneous glycosylation events [46].

Representative applications. Sulfoglycolipid synthesis: The Boons research group utilized preactivation strategies to construct core tetra- and pentasaccharides bearing orthogonal protecting groups (Alloc, Lev, TBS). Through sequential deprotection and sulfation steps, the team successfully synthesized functionalized sulfoglycolipids capable of participating in cellular signaling pathways [48].

Preparation of extended glycan structures: Researchers led by Ye employed preactivation-based synthetic strategies to construct two landmark carbohydrates: (1) a linear 1080-unit polysaccharide, representing the longest synthetic oligosaccharide reported to date [49,50]; and (2) a structurally complex 92-unit mycobacterial arabinogalactan, that is essential for preserving the structural stability of the mycobacterial cell wall [8]. These achievements underscore the robustness and scalability of the methodology for synthesizing glycans with high architectural complexity.

### 2.4. Linear and Convergent Block Strategy

In carbohydrate chemistry, two conventional strategies for the construction of oligosaccharides are widely recognized: linear assembly and convergent block synthesis. The linear approach involves the stepwise addition of monosaccharide units, while the convergent method entails the coupling of pre-formed oligosaccharide segments. The block coupling is especially advantageous in the synthesis of complex carbohydrate chains, as it minimizes repetitive glycosylation steps and significantly improves synthetic efficiency when constructing extended molecular architectures [51,52].

Linear synthetic approaches: It involves the stepwise assembly of glycosyl donors and acceptors through iterative coupling reactions, in which intermediate products are chemically transformed into reactive species to enable chain elongation. Although this strategy requires protective group manipulations between synthetic steps, it remains widely employed for the synthesis of short to moderately long oligosaccharides due to its operational simplicity.

**Figure 6:**
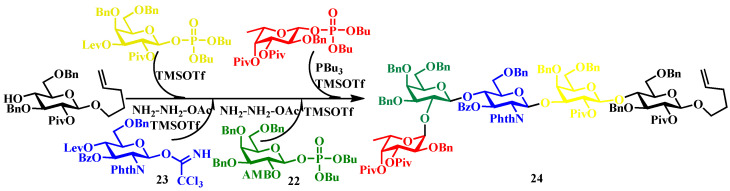
Synthesis of linear oligosaccharides via alternating glycosylation.

Representative applications. The blood-group determinant H-type II pentasaccharide **24** synthesis [53]: In this synthesis, galactosyl phosphate **22** was reacted with acceptor **23** in the presence of TMSOTf as an activator. Subsequent deprotection of the levulinoyl (Lev) group allowed sequential glycosylations using trichloroacetimidate (TCAI) donors bearing Lev and allyl methoxybenzyl (AMB) protecting groups, ultimately furnishing the target pentasaccharide in 60% overall yield (Figure 6) [53]. Recent advancements, including solid-phase synthesis [54] and fluorous-tag-assisted strategies [55], have further enhanced the efficiency of purification without compromising synthetic performance.

Convergent Block Assembly. This strategy involves the preparation of pre-assembled carbohydrate modules, such as disaccharide or trisaccharide units, which are subsequently coupled to construct target oligosaccharides [51,52]. By reducing the number of required glycosylation steps and minimizing error accumulation during synthesis, this method demonstrates superior efficiency in assembling complex branched architectures and large-scale carbohydrate structures.

Key Challenges. The strategic incorporation of challenging glycosidic linkages-such as α-sialyl bonds-during the early stages of the synthetic sequence further enhances overall yield and selectivity compared to conventional linear approaches [52].

Representative applications. Case Study 1: Synthesis of Ganglioside GP3 [56]. Kiso’s group implemented a [2 + 2] convergent strategy, coupling two tetrasaccharide precursors at 0 °C under NIS/TfOH activation to afford an octasaccharide intermediate in 91% yield. Subsequent protection with trichloroacetyl (TCAI) enabled efficient ligation with a ceramide acceptor via TMSOTf-catalyzed glycosylation, leading to the successful assembly of GP3 in 77% yield [56]. Various oligosaccharides have been synthesized through convergent assembly [57,58,59]. Case Study 2: Construction of Mycobacterial Arabinogalactan. Ye’s research team has reached a pinnacle with the creation of a substantial mycobacterial arabinogalactan oligosaccharide consisting of 92 monosaccharide units (92-mer) [8].

### 2.5. Programmable One-Pot Synthesis Strategy

This approach integrates computer-aided design methodologies with one-pot glycosylation techniques, enabling accelerated and automated oligosaccharide production, as evidenced by recent studies [32,60]. This innovative strategy is grounded in enzymatic one-pot principles [9,61,62,63] and comprises two core components: (1) a comprehensive repository of glycosyl donors with experimentally validated relative reactivity values (RRVs), and (2) specialized route-planning algorithms that systematically optimize synthetic pathways according to the target carbohydrate structures

Critical Elements. Donor Repository: Researchers have systematically cataloged RRVs for over 400 thioglycoside donors, the most widely used class in programmable glycosylation systems-encompassing a broad range of protective group configurations [32]. The preference for thioglycosides is attributed to their high chemical stability, ease of synthesis, and compatibility with various activation conditions. [60].

OptiMer: Developed by Zhang et al. in 1999, this computational tool maintains a comprehensive database containing relative reactivity values (RRVs) for thioglycoside donors and recommends optimal building block combinations for oligosaccharide synthesis. Furthermore, the system predicts α/β-stereochemical outcomes by analyzing protective group configurations [60].

Auto-CHO: Enhanced by the improvements introduced by Cheng et al. (2018), this platform enables hierarchical one-pot synthetic strategies, including modular block coupling operations. It utilizes predictive machine learning models to estimate relative reactivity values (RRVs) for novel donor molecules [32], integrating 154 experimentally validated building blocks with approximately 50,000 computationally derived virtual building blocks (BBLs) that incorporate predicted reactivity parameters [32].

**Figure 7:**
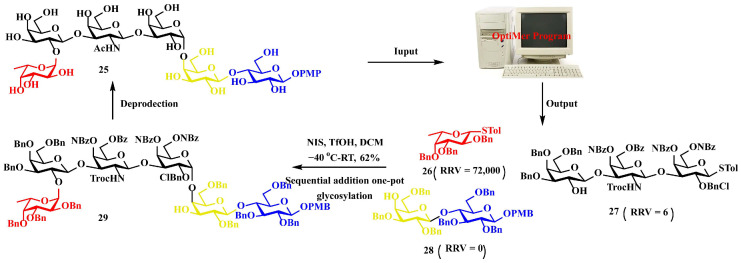
The OptiMer computational platform facilitates the programmable one-pot assembly of Globo H carbohydrate structures.

Application in Globo H hexasaccharide assembly. The tumor-associated carbohydrate antigen Globo H hexasaccharide **25** serves as a critical component in prostate cancer immunotherapy. Computational analysis using OptiMer identified optimal building blocks **26**–**28** and proposed an efficient one-pot synthetic pathway (Figure 7) [64,65]. By employing stepwise activation of glycosyl donors guided by relative reactivity values, followed by strategic protective group manipulations, the complex oligosaccharide structure was successfully assembled, demonstrating the potential of algorithm-driven approaches in the synthesis of therapeutic carbohydrates [10,66,67].

### 2.6. Solid-Phase Synthesis Strategy

Linear solid-phase synthesis (SPS) involves immobilizing the growing glycan chain onto a polymeric support, such as functionalized beads, thereby enabling automated synthesis and eliminating the requirement for intermediate purification steps [68,69]. This approach is based on three essential components: (1) Anchoring systems: These covalently attach carbohydrates to the solid support via cleavable linkers, allowing for controlled release of the final product-for example, through photolabile groups, traceless anchors, or macromolecular spacers [68,70,71,72,73]. (2) Molecular spacers: These alleviate steric hindrance caused by the proximity to the solid matrix and improve the efficiency of molecular interactions [68]. (3) Programmable instrumentation: Automated platforms perform sequential reactions, including sugar coupling, deprotection, and washing cycles, under computer-controlled protocols, significantly minimizing manual handling [50,69].

**Figure 8:**
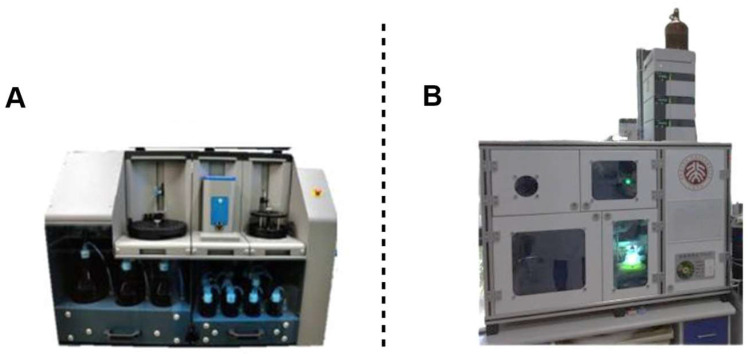
Automatic instrumentation. (**A**) Automation glyconeer 2.1^TM^; (**B**) Glycan automated synthesizer developed by the Ye group.

Automated glycan assembly (AGA) represents the most advanced approach in solid-phase oligosaccharide synthesis, leveraging robotic platforms to enable precise and efficient construction of complex carbohydrate structures [74,75]. Key technological advancements in this domain include: (1) Cis-glycosidic bond formation: The incorporation of monosaccharides functionalized with non-adjacent protecting groups—such as acetyl and benzoyl derivatives—has facilitated the stereoselective synthesis of cis-configured glycosidic linkages, including α-mannosides [69]. Integration of robotic automation: The commercially available Glyconeer 2.1™ platform (Figure 8A) automates critical synthetic steps, such as coupling reactions, deprotection, and final product cleavage, thereby significantly improving the efficiency and reproducibility of synthesizing structurally intricate glycans [69]. This methodology has been successfully employed by Budhadev and colleagues in the synthesis of heparan sulfate precursors exhibiting potential anticoagulant activity [70,76,77].

Solution-phase automation. Ye’s research group developed an automated dual-mode solution-phase system for glycan assembly (Figure 8B) based on preactivation strategies and iterative amplification techniques [50]. This innovative platform overcomes critical limitations of conventional solid-phase methods, particularly spatial constraints, and has enabled the synthesis of exceptionally long oligosaccharide chains up to a 1080-mer [50], exceeding the maximum lengths achieved in artificially synthesized nucleic acids (200-mer) and polypeptides (472-mer) [78].

## 3. Conclusions

Oligosaccharides are involved in numerous biological functions and have significant applications in pharmaceutical applications; however, their chemical synthesis is hindered by complex structural characteristics and stringent stereochemical requirements. This review evaluates six fundamental synthetic strategies: One-pot synthesis strategy, orthoganal protection strategy, preactivation strategy, linear and convergent block strategy, programmable one-pot synthesis strategy, and solid-phase synthesis strategy. Each method exhibits distinct advantages for specific applications. The one-pot synthesis strategy demonstrates high efficiency in the synthesis of oligosaccharides of intermediate length. Orthogonal protection strategy is particularly effective for constructing branched structures or molecules incorporating rare monosaccharide units. Preactivation strategy enables the assembly of exceptionally long polysaccharide chains, including polymers comprising up to 1080 monosaccharide units. The convergent block strategy is well-suited for synthesizing large oligosaccharides, for instance, the 92-unit mycobacterial arabinogalactan. Programmable one-pot synthesis strategy leverage machine learning to optimize synthetic route design and enhance planning efficiency. Solid-phase synthesis strategy facilitates automated and scalable production through iterative coupling processes, making them ideal for industrial-scale manufacturing.

The evolution of oligosaccharide synthesis is expected to advance through three principal directions. First, catalytic innovation: advances in catalyst design will focus on developing regioselective and stereospecific glycosylation catalysts, significantly reducing the reliance on protective group strategies. Second, computational integration: the application of artificial intelligence will enable the construction of predictive models for glycosylation reactions and the optimization of synthetic routes, as exemplified by platforms such as Auto-CHO’s machine learning framework. Third, biomedical implementation: translational efforts will prioritize the large-scale production of glycan-based vaccines, such as Globo-H, and therapeutic agents, including anti-inflammatory heparan sulfate analogs, to meet clinical needs. These advancements are poised to democratize access to oligosaccharide synthesis, thereby accelerating progress in glycostructure research and fostering the development of next-generation carbohydrate-based therapeutics.

## Data Availability

Not applicable.
